# Sleep quality and associated factors among the elderly living in rural Chiang Rai, northern Thailand

**DOI:** 10.4178/epih.e2018018

**Published:** 2018-05-14

**Authors:** Weerakorn Thichumpa, Nopporn Howteerakul, Nawarat Suwannapong, Visasiri Tantrakul

**Affiliations:** 1 Department of Epidemiology, Faculty of Public Health, Mahidol University, Bangkok, Thailand; 2 Department of Public Health Administration, Faculty of Public Health, Mahidol University, Bangkok, Thailand; 3 Department of Medicine, Faculty of Medicine Ramathibodi Hospital, Mahidol University, Bangkok, Thailand

**Keywords:** Sleep hygiene, Elderly, Prevalence, Depression, Family relations

## Abstract

**OBJECTIVES:**

This study aimed to characterize the prevalence of poor sleep quality and to identify associated factors among community-dwelling elderly individuals in northern Thailand.

**METHODS:**

A cross-sectional study was conducted among 266 randomly selected elderly people in a sub-district in rural Chiang Rai Province, northern Thailand. The participants were interviewed using the Thai version of the Pittsburgh Sleep Quality Index (PSQI).

**RESULTS:**

Roughly 44.0% of the participants had poor sleep quality (PSQI score, >5), 9.4% used sleep medication, 27.1% had poor family relationships, and 12.0% had mild depression. Multiple logistic regression analysis indicated that being female (odds ratio [OR], 1.74; 95% confidence interval [CI], 1.10 to 3.02), a higher education level (OR, 3.03; 95% CI, 1.34 to 6.86 for primary school; OR, 2.48; 95% CI, 1.31 to 5.44 for higher than primary school), mild depression (OR, 2.65; 95% CI, 1.11 to 6.36), and poor family relationships (OR, 3.65; 95% CI, 1.98 to 6.75) were significantly associated with poor sleep quality.

**CONCLUSIONS:**

The prevalence of poor sleep quality among the elderly was moderately high. Healthcare providers should regularly conduct screenings for sleep quality and depression; provide sleep health education; and conduct interventions to encourage participating in family activities, resolving conflicts, sharing ideas, and making compromises within the family.

## INTRODUCTION

Poor sleep quality is a common problem in the general population, often due to work-related demands and lifestyle changes [1- 3]. Problems with sleep increase sharply with age [4-9]. Poor sleep quality is associated with serious negative consequences on physical, mental, and social aspects of well-being [10]. These effects are often more pronounced in individuals who are living with multiple chronic diseases such as cancer, stroke, and the decreasing performance associated with aging [6,10,11]. Poor sleep quality can have profound physical effects on the elderly, including fatigability and an increased risk of falls. These effects threaten both mobility and independence [3,4,7]. Poor sleep quality and sleep deprivation are also associated with effects on activities of daily living and cognitive impairment in the elderly [12,13]. Moreover, both are significantly related with psychological problems, mood changes, and depression [1,7,14,15]. Previous studies have indicated that supportive ties and close relationships among family members are positively related to good sleep quality, while aversive ties predict poor sleep quality [16,17].

The prevalence of poor sleep quality varies according to the demographic characteristics of the population. In Australia, epidemiological surveys have found that roughly 13.0-33.0% of elderly people reported frequent sleep difficulties or problems maintaining sleep [18]. In China, Li et al. [8] reported that the prevalence of poor sleep quality among the elderly in rural Anhui Province was 49.7%. In that study, the associated factors included age, physical and mental health, and nutritional intake. In Korea, Park et al. [15] reported that approximately 60.0% of the elderly aged 65 years and above, sampled from 5 community health centers in Gyeonggi Province and Seoul, reported having poor sleep quality. In Thailand, epidemiologic data from 1997 to 2015 indicated that the prevalence of poor sleep quality ranged from 42.7 to 57.1%, and that the factors leading to sleep disturbances were pain, anxiety, depression, and poor perceived health [19-21]. A more recent study by Aunjitsakul et al. [22] revealed that the prevalence of poor sleep quality among the elderly seeking care at the outpatient department of a teaching hospital in a southern province of Thailand was 70.8%. The main complaints were short sleep duration (99.7%), poor sleep efficiency (61.6%), and daytime dysfunction (51.6%). However, that study did not identify any factors associated with poor sleep quality in its multivariable analysis. The cited studies used different types of sleep quality measures, different definitions of older adults (some used 60 years of age as a cutoff for inclusion, while others used 65 years), and varied in terms of study location, including urban, rural, and hospital settings. This should be kept in mind when comparing the prevalence rates of poor sleep quality in older adults.

The proportion of the elderly in Thailand has been steadily increasing. The number of people over 60 is projected to increase from 10.73 million in 2015 to 18.36 million in 2030, when this age group will account for 26.9% of the total population [23].

Wiang Ka Long is a sub-district in rural Chiang Rai Province. Demographic data from the Chiang Rai Provincial Health Office show that the proportion of the elderly (age 60 and above) in this sub-district has increased from 18.9% in 2005 to 24.9% in 2015, and will continue to increase quickly in future decades [24]. In Chiang Rai Province, the 5 leading diseases of the elderly are hypertension, diabetes mellitus, ischemic heart disease, stroke, and chronic obstructive pulmonary disease [25]. All these chronic diseases are associated with poor sleep quality [26,27]. Previous studies have also found sleep problems to be common among the elderly, to the point that many elderly individuals perceive sleep problems as part of the normal aging process [28,29]. Therefore, sleep problems in the elderly are frequently underexplored and untreated [15,30]. In Thailand, information about sleep quality among the community-dwelling elderly is still scarce, particularly for those living in rural areas. We conducted a study examining the prevalence of poor sleep quality and its associated factors using a localized version of the Pittsburgh Sleep Quality Index (PSQI) [31,32] to help fill this information gap. The results of our study can be used to identify appropriate interventions to promote the physical and mental health of the elderly with poor sleep quality.

## MATERIALS AND METHODS

### Study participants

**Figure 1.**
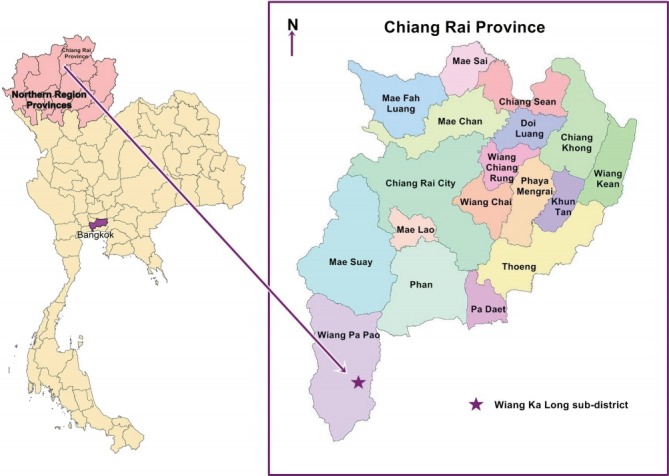
Map showing Wiang Ka Long sub-district.

This cross-sectional study was conducted in the Wiang Ka Long sub-district, in rural Chiang Rai Province in northern Thailand. Wiang Ka Long is 785 km away from Bangkok, the capital of Thailand (Figure 1). This sub-district was selected because of its high proportion of elderly inhabitants. In 2015, the elderly accounted for 23.9% (1,618 of 6,751) of the population [24]. The sample size was estimated using the single-proportion formula with finite population correction [33] and a 95% confidence interval (CI). The sample size calculation was based on 46.3% of elderly Thais reporting insomnia in a previous epidemiological survey [20]. Precision was set at 5.5%, and the sample size was calculated to be 266 to represent the population of 1,618 elderly inhabitants [24]. A total of 279 elderly individuals were recruited to allow for a nonresponse rate of approximately 5.0%. The sub-district has 15 villages, and 17-20 households per village were randomly selected. The participants were selected using probability proportional to size sampling, to obtain study samples that represented each village and age group (60-69, 70-79, and ≥ 80 years). If no one was home, the nearest household was selected. When a household had more than 1 eligible elderly member, an elderly household member was chosen at random. The inclusion criteria were being a registered resident of the study area, being aged ≥ 60 years, having no problems hearing and speaking, and being willing to participate in the study. The elderly who were unavailable for an interview during the study or those who the research assistant determined, through interacting with them, to be too ill or cognitively impaired to participate were excluded. Of the 279 participants, 13 were excluded because their questionnaires were incomplete. Therefore, responses from 266 participants were analyzed.

### Instrument and measurements

The research instruments of this study consisted of 5 parts.

General characteristics included age, sex, education, occupation, religious activities, chronic diseases, daytime napping, use of sleep medication, alcohol consumption, smoking, and perceived environmental factors disturbing sleep quality. These variables have been reported to be associated with poor sleep quality in previous studies [4,7,15,19-21,26,27,34,35].

Depression was measured using the Thai version of the Patient Health Questionnaire [36,37], which contains 9 questions used to screen for depression. Each question is rated on scale of 0 to 3, and the total score is 27 points. A score of < 7 is defined as no depressive symptoms, 7-12 as mild depression, 13-18 as moderate depression, and 19-27 as severe depression. The Cronbach alpha of this instrument was found to be 0.92.

Activities of daily living were assessed with the modified Barthel Index [38,39], which comprises 10 questions used to assess hygiene, bowel control, bladder control, toileting, feeding, moving, ambulation, chair transfer, dressing, stair climbing, and bathing oneself. The total score is 20. A score of 0-4 is defined as dependence, 5-11 as some dependence, and 12-20 as independence. The Cronbach alpha was 0.95.

Family relationships were measured by the Family Relationships Scale developed by the Technical Promotion and Support Offices of the Thai Department of Social Welfare [40]. This instrument comprises 10 questions with a 3-point response format: 0 (never), 1 (sometimes), and 2 (always). Scores of > 10 are defined as good relationships, and scores ≤ 10 as poor relationships. The Cronbach alpha was 0.95.

Sleep quality was examined using the Thai version of the Pittsburgh Sleep Quality Index (T-PSQI) [31,32], a self-rated questionnaire used to assess subjective sleep quality and sleep disturbance over 1 month. It contains 19 items, which generate 7 component scores: subjective sleep quality (1 item), sleep latency (2 items), sleep duration (1 item), habitual sleep efficiency (3 items), sleep disturbance (9 items), use of sleep medication (1 item) and daytime dysfunction (2 items). Each component has a range of 0-3 points, ranging from 0 (no difficulty) to 3 (severe difficulty) [41]. The totals of the 7 component scores are added to generate the final score (range, 0-21). Higher scores indicate poorer subjective sleep quality. Habitual sleep efficiency was calculated by: hours of sleep/(get up time – usual bed time)× 100. Sleep disturbance was calculated from the T-PSQI items ‘wake up in the middle of the night or early morning,’ ‘need to get up to use the bathroom,’ ‘cannot breathe comfortably,’ ‘cough or snore loudly,’ ‘feel too cold,’ ‘feel too hot,’ ‘have bad dreams,’ and ‘have pain.’ Higher scores represent more sleep disturbances. Daytime dysfunction was calculated from the T-PSQI items ‘during the past month, how often have you taken medicine to help you sleep (prescribed or over the counter)’ and ‘during the past month, how often you have you had trouble staying awake while driving, eating meals, or engaging in social activity.’ Higher scores represent more daytime dysfunction. Participants with a T-PSQI global score of > 5 were defined as having poor sleep quality, while T-PSQI global scores of ≤ 5 were defined as good sleep quality. The Cronbach alpha was 0.84 [32].

### Data collection

Data were collected by the principal investigator and 2 trained research assistants between June and September 2016. Written informed consent was obtained from all participants. The research team explained the objectives of the study and interviewed the participants using structured questionnaires.

### Statistical analysis

Data were analyzed using SPSS version 18.0 (SPSS Inc., Chicago, IL, USA). Descriptive statistics such as percentage, mean, and standard deviation (SD) were used to describe all study factors. Multiple logistic regression was used to obtain odds ratios (ORs) and 95% CIs to examine the associations between factors analyzed in the study and poor sleep quality. In the analysis, sleep quality was dichotomized into good sleep quality and poor sleep quality. All study factors with a p-value < 0.05 in the univariate analysis and biological plausibility were tested for the absence of multicollinearity among study factors by inspecting the magnitude of the standard error (SE) of each variable. The magnitude of the SEs should hover around 0.001-5.0 [42]. In model I, the predictors were adjusted for age, sex, and education level. In model II, the predictors were adjusted for age, sex, education level, mild depression, and family relationships. The p-values < 0.05 were considered to indicate statistical significance.

### Ethical approval

The study protocol was approved by the ethics committee of the Faculty of Public Health, Mahidol University (COA. no. MUPH 2016-086; June 7, 2016).

## RESULTS

### General characteristics

Of the 266 participants, 59.4% were female, and 56.4% were aged 60-69 years. The mean± SD age was 67.8± 7.1 years. Moreover, 52.2% had finished primary school or higher, 47.4% were not working or a housewife, 34.2% were agricultural laborers, and 9.8% were self-employed. Religious activities were performed by 95.9% of the participants. Of the respondents, 59.8% had at least 1 chronic disease; 38.2% had hypertension, and 14.7% had diabetes. Furthermore, 22.2% often napped in the daytime, 9.4% used sleep medication, 19.2% reported that they sometimes or often drank alcohol, and 10.1% reported that they sometimes or often smoked. The environmental factors reported as disturbing sleep quality were noise (12.4%), feeling too hot (6.0%), and light (3.8%), as shown in Table 1.

### Activities of daily living, depression, and family relationships

As shown in Table 2, only 1.1% of the participants reported they had some dependence on others for daily living activities. The mean Barthel Index mean±SD score was 19.7±1.4. Symptoms of mild depression were shown in 12.0% of the participants. The mean±SD depression score was 2.6±2.7. Of the participants, 27.1% had poor family relationships. The mean family relationship mean±SD score was 14.6±2.2.

### Sleep quality

The mean± SD T-PSQI score was 5.86± 2.47. The prevalence of poor sleep quality (T-PSQI global score, > 5) was 44.0%. The mean± SD sleep duration was 7.0± 1.3 hours. The mean± SD sleep latency was 30.0± 27.5 minutes. The mean± SD sleep efficiency among good sleepers was 92.6± 7.7%, while it was 83.7± 16.6% among poor sleepers. The mean T-PSQI score of participants with at least 1 chronic disease or mild depression was slightly higher than that of participants without these conditions (5.02 vs. 4.63 for chronic disease and 6.25 vs. 4.68 for depression), as shown in Table 3.

Table 4 shows the number and percentage of poor sleepers (PSQI > 5) by sleep quality components and items. Among the 117 poor sleepers, 14.5% reported poor subjective sleep quality, 86.3% reported sleep latency of more than 15 minutes, 23.1% reported a good or recommended sleep duration (> 7 hours), 59.8% reported a low but possibly appropriate sleep duration (6-7 hours), 41.9% had poor sleep efficiency (< 85.0%), 82.1% reported frequent sleep disturbances (≥ 1/wk), 18.0% used sleep medication (< 1/ wk or ≥ 1/wk), and 35.9% reported daytime dysfunction (< 1/wk or ≥ 1/wk).

Table 5 depicts the details of reported sleep disturbance problems (> 1/wk) among poor sleepers. The most frequently reported sleep problem was needing to get up to use the bathroom (71.8%), followed by waking up in the middle of the night or early morning (64.9%) and difficulty falling asleep within 30 minutes (57.3%).

### Factors associated with poor sleep quality

In the univariate analysis, 3 factors were significantly associated with poor sleep quality among the elderly: a higher education level (OR, 2.72; 95% CI, 1.31 to 5.64 for primary school; OR, 1.83; 95% CI, 1.02 to 3.50 for higher than primary school), mild depression (OR, 3.22; 95% CI, 1.46 to 7.11), and poor family relationships (OR, 3.95; 95% CI, 2.22 to 7.03).

In the multivariate analysis, age, sex, education level, and all other variables found to be significant in the univariate analysis were simultaneously included in the final analysis. In model I, the significant predictors were being female (OR, 1.90; 95% CI, 1.12 to 3.21), higher education level (OR, 3.13; 95% CI, 1.45 to 6.74 for primary school; OR, 2.22; 95% CI, 1.06 to 4.63 for higher than primary school). The Hosmer and Lemeshow goodness-of-fit test suggested the model was a good fit with the data, with p= 0.164. In model II, the significant predictors were being female (OR, 1.74; 95% CI, 1.10 to 3.02), higher education level (OR, 3.03; 95% CI, 1.34 to 6.86 for primary school; OR, 2.48; 95% CI, 1.31 to 5.44 for higher than primary school), mild depression (OR, 2.65; 95% CI, 1.11 to 6.36), and poor family relationships (OR, 3.65; 95% CI, 1.98 to 6.75). The Hosmer and Lemeshow goodness-of-fit test also suggested a good fit with the data (p= 0.218), as shown in Table 6.

## DISCUSSION

The results of this study revealed that the prevalence of poor sleep quality among the elderly in the study area was 44.0%. The mean± SD T-PSQI global score was 5.86± 2.47. This rate is comparable with the prevalence of 42.7% found in a 1997 study of Thai community-dwelling elderly in the Nong Chok district of Bangkok reported by Sukying & Ninchaikovit [19]. Although Nong Chok is now an urban area, in 1997 it was rural and agriculture was the main economic activity, making it similar to the presentday Wiang Ka Long sub-district. An epidemiological study by Sukying et al. [20] on insomnia among 40,111 individuals aged 60 years and above from 4 regions of Thailand found the prevalence of poor sleep quality to be 46.3%. Choombuathong et al. [21] and Aunjitsakul et al. [22] reported higher prevalence rates (57.1 and 70.8%, respectively) than that found in the current study. This is most likely because these studies examined sleep quality among the elderly in very different contexts (a nursing home and an outpatient department) from that of the current study.

When the proportions of poor sleep quality components/items among the 117 poor sleepers were reviewed, the 2 most frequent complaints were poor sleep latency (86.3%) and frequent sleep disturbances (82.1%). This finding is congruent with previous studies [7,23] that found the most frequently occurring sleep problem in elderly was poor sleep latency, which occurs when people may spend some hours in bed before falling asleep. In our study, 57.3% (67 of 117) of the poor sleepers reported having difficulty falling asleep within 30 minutes. Within the category of sleep disturbances, 71.8% (84 of 117) of the poor sleepers reported getting up to use the bathroom and 64.9% (76 of 117) reported waking up in the middle of the night or early morning. This finding is consistent with the studies by Knodel et al. [23] and Barthlen [44]. The proportion of poor sleepers getting a good or recommended sleep duration (> 7 hours) was low (23.1%). Moreover, 64.9% (76 of 117) of the poor sleepers and 70.5% (105 of 149) of the good sleepers napped in the daytime. Daytime napping might alleviate some of the problems with poor sleep duration in both good and poor sleepers. It would be beneficial to investigate the influence of daytime napping on nocturnal sleep in the community-dwelling elderly aged 60 years and above.

In the multivariate analysis, sex, education level, mild depression, and family relationships were significant predictors of sleep quality. In model I, sex changed from a nearly significant predictor (p= 0.059) in the univariate analysis to a significant predictor (p< 0.05) of sleep quality. This was because, as we found through subgroup and stratification-based analyses, more females than males had finished primary school or higher (56.8 vs. 43.2%), had mild depression (15.2 vs. 7.4%), and reported poor family relationships (27.8 vs. 25.9%). In addition, the mean T-PSQI score for females was higher than for males (6.15 ± 2.51 vs. 5.44 ± 2.09). These results suggest that sex alone did not account for sleep quality. Instead, it was partially influenced by other variables in the model. This finding is consistent with that of Sukying et al. [20].

In this study, a higher level of education (completion of primary school) was associated with poor sleep quality. This may have been due to occupational stress, since some of the elderly with a higher level of formal education were still working. This is in line with the results of Yao et al. [45]. However, this result does not agree with the finding of Luo et al. [4] who found that Chinese elderly in Shanghai with less education had poorer sleep quality than those with more education. Possible reasons for this discrepancy include differences between the studies regarding the mean years of education and study location. In the Shanghai study, the participants’ education level was higher than that of the participants in the present study (11.0±4.5 vs. 3.0±2.3 years). In addition, the greater earning capacity of the elderly who live in urban areas may lead to a reduction in some of the factors that cause low-quality sleep. Additionally, the earning gap between the education levels of ‘some primary school’ and ‘completed primary school’ in rural areas is unlikely to be dramatic, but the earning gap between the levels of ‘completed primary school’ and ‘completed university’ in an urban setting is likely to be large.

Mild depression was an important predictor of sleep quality, consistent with previous studies [14,45]. The prevalence of depression in Thailand has been reported to range from 5.9 to 38.9% [46-48] depending on the study population and the instrument used in the study. Females have been shown to be more likely to have depression than males [48,49]. Depression can have several impacts on daily activities, emotional well-being, and the risk of suicide [50]. Therefore, healthcare providers should pay more attention to sleep habits in the elderly. Appropriate depression screening, access to mental health care, and interventions to manage depression might be able to prevent further potential negative effects related to depression in the elderly.

A poor family relationship was the most significant predictor of sleep quality in this study. This finding is consistent with those of the study by Ailshire & Burgard [16], who found that having a strained family relationship was associated with more troubled sleep, whereas a supportive family relationship was associated with less troubled sleep. Responses to 4 items in the family relationship scale revealed that 28.6% of the elderly reported never spending a significant amount of time participating in family activities such as reading, watching TV, and listening to the radio; 21.8% reported never resolving conflicts or consulting other family members before making a decision; 21.1% reported never sharing ideas among family members; and 20.7% never experienced good compromises among family members (data not shown). Given the strong relationship between the quality of family relationships and the quality of sleep, healthcare providers should provide interventions that promote good relationships among family members. Good family relationships can be a buffer against the loneliness and depression that affect sleep quality in the elderly [17].

This study had 4 main limitations that should be considered when interpreting its findings. First, the results can be generalized only to the study area due to the power of the sample size calculation. Second, sleep quality was measured by self-reporting only and no objective measures, such as a polysomnography test, were included. Third, this study focused on family relationships, which were found to be a major factor affecting sleep quality in the elderly in previously published studies [16,17,44]. However, some elderly persons might have poor relationships with non-family members, such as friends or neighbors, that might affect their sleep quality. This possibility was not investigated in the present study. Fourth, the cross-sectional study design limits the degree to which conclusions can be drawn regarding the causal relationship between the factors analyzed in this study and sleep quality among the elderly in the study area. To increase the generalizability of the findings, this study should be replicated with a greater sample size and larger study area.

Our findings indicated that the prevalence of poor sleep quality among the elderly in the study area was high, and the major significant predictors of poor sleep quality were mild depression and poor family relationships. Given the impact of poor sleep quality on health, healthcare providers should regularly screen for sleep quality and depression and provide interventions targeting sleep health education and behavior change. Educational messages should include recommending that the elderly go to bed at the same time each night and wake up at the same time each morning, and that they should make their bedroom environment quiet, dark, and not too hot or cold. Further interventions could include providing consultations to encourage family members to participate in activities with their elderly family members, as well as providing informal training on resolving conflicts, sharing ideas, and finding compromises. Psychosocial interventions with a non-pharmacological approach, such as community exercise programs, should be also introduced to improve sleep quality and reduce depression among the elderly in the community.

## Figures and Tables

**Table 1.** General characteristics of the subjects (n=266)

Variable	n (%)
Sex	
Male	108 (40.6)
Female	158 (59.4)
Age (yr)	
60-69	150 (56.4)
70-79	83 (31.2)
≥ 80	33 (12.4)
Mean±SD (range)	67.8±7.1 (60-85)
Education level	
No school	59 (22.2)
Primary school (grade 4 intheformer Thai educational system)	68 (25.6)
>Primary school (higher than grade 4)	139 (52.2)
Occupation	
None/housewife	126 (47.4)
Agriculture	91 (34.2)
Self-employed	26 (9.8)
Employee	20 (7.5)
Others	3 (1.1)
Religious activities (e.g., alms-giving, listening to sermons, meditation)	
Yes	255 (95.9)
No	11 (4.1)
Have 1 or more chronic diseases (hypertension, diabetes, etc.)	159 (59.8)
Type of chronic disease^1^	
Hypertension	102 (38.2)
Diabetes	39 (14.7)
Arthritis	23 (8.6)
Heart disease	10 (3.8)
Others (cancer, kidney disease, hypercholesterolemia, etc.)	82 (30.8)
Daytime napping	
No	85 (31.9)
Sometimes	122 (45.9)
Often	59 (22.2)
Use of sleep medication	25 (9.4)
Alcohol consumption	
Never or no longer drink	215 (80.8)
Sometimes or often drink	51 (19.2)
Smoking	
Never or no longer smoke	239 (89.8)
Sometimes or often smoke	27 (10.1)
Reported environmental factors disturbing sleep quality^1^	
None	186 (69.9)
Noise	33 (12.4)
Feeling too hot	16 (6.0)
Too much light	10 (3.8)
Feeling too cold	5 (1.9)
Other (e.g. uncomfortable mattress, insects, bad smells)	22 (8.3)

SD, standard deviation.

1 Multiple responses.

**Table 2.** ADLs, depression, and family relationships among the subjects

Variable	n (%)
ADLs (BI score)	
Independence (12-20)	263 (98.9)
Some dependence (5-11)	3 (1.1)
Dependence (0-4)	0 (0.0)
Mean±SD (range)	19.7±1.4 (6-20)
Depression (score)	
Mild (7-12)	32 (12.0)
None (<7)	234 (88.0)
Mean±SD (range)	2.6±2.7 (0-12)
Family relationships (score)	
Good (≥10)	194 (72.9)
Poor (<10)	72 (27.1)
Mean±SD (range)	14.6±2.2 (0-20)

ADL, activities of daily living; BI, Barthel Index; SD, standard deviation.

**Table 3.** The T-PSQI global score among the subjects according to the presence ofchronic diseases and depression

Variables	Mean± SD
No. of chronic diseases	
None	4.63±2.26
One	5.02±2.45
Two or more	5.01±2.45
Depression (score)	
None (<7)	4.68±2.24
Mild depression (7-12)	6.25±2.82

T-PSQI, Thai version of the Pittsburgh Sleep Quality Index; SD, standard deviation.

**Table 4.** Good sleepers and poor sleepers among the subjects by sleep quality componentsand items^1^

Sleep quality componentsand items	Good sleeper	Poor sleeper	Total (n=266)
	PSQI≤5 (n=149)	PSQI>5 (n=117)	
Subject sleep quality			
Good (0, 1)	149 (100.0)	100 (85.5)	249 (93.6)
Poor (2, 3)	0 (0.0)	17 (14.5)	17 (6.4)
Sleep latency (min)^2^			
Good (≤15) (0)	77 (51.7)	16 (13.7)	93 (35.0)
Poor (>15) (1-3)	72 (48.3)	101 (86.3)	173 (65.0)
Sleep duration (hr)			
Good or recommended (>7) (0)^3^	119 (79.9)	27 (23.1)	146 (54.9)
Low, may be appropriate (6-7) (1)^3^	0 (0.0)	70 (59.8)	70 (26.3)
Poor (≤6) (2, 3)	30 (20.1)	20 (17.1)	50 (18.8)
Habitual sleep efficiency (%)^4^			
Good (≥85) (0)	127 (85.2)	68 (58.1)	195 (73.3)
Poor (<85) (1-3)	22 (14.8)	49 (41.9)	71 (26.7)
Sleep disturbance (times/wk)^5^			
Low (<1 or not during past month) (0, 1)	115 (77.2)	21 (17.9)	136 (51.1)
High (≥1) (2, 3)	34 (22.8)	96 (82.1)	130 (48.9)
Use of sleep medication			
Never in 1 month (0)	145 (97.3)	96 (83.1)	241 (90.6)
<1/wk or ≥1/wk (1-3)	4 (3.4)	21 (18.0)	25 (9.4)
Daytime dysfunction^6^			
Never in 1 month (0)	135 (90.6)	75 (64.1)	210 (78.9)
<1/wk or ≥1/wk (1-3)	14 (9.4)	42 (35.9)	56 (21.1)

Values are presented as number (%). PSQI, Pittsburgh Sleep Quality Index.

1 Scores range from 0 to 3; 0 indicates no difficulty and 3 indicates severe difficulty.

2 The proportion of the first item of the sleep latency component was presented to make it easier to understand.

3 Defined according to the National Sleep Foundation [43].

4 Habitual sleep efficiency was calculated as: hours of sleep/(get up time – usual bed time)×100.

5 Derived from PSQI items 5b-5j; higher scores indicate more sleep disturbance; item 5j is an open-ended question for describing trouble sleeping. It was not included in the analysis, as there were no responses.

6 Derived from PSQI items 7-8; higher scores indicate more daytime dysfunction.

**Table 5.** Reported sleep disturbances among 117 poor sleepers (PSQI>5)

Sleep disturbance (>1/wk)	n (%)
Cannot fall asleep within 30 minutes	67 (57.3)
Wake up in the middle of the night or early morning	76 (64.9)
Need to get up to use the bathroom	84 (71.8)
Cannot breathe comfortably	5 (4.3)
Cough or snore loudly	30 (25.6)
Feel too cold	5 (4.3)
Feel too hot	16 (13.6)
Have bad dreams	9 (7.7)
Have pain	20 (17.1)

PSQI, Pittsburgh Sleep Quality Index.

**Table 6.** Factors associated with poor sleep quality among the subjects

Variable	Crude OR (95%CI)	Model I^1^ aOR (95%CI)	Model II^2^ aOR (95% CI)	p-value
Age (yr)				
60-69	1.00 (reference)	1.00 (reference)	1.00 (reference)	
70-79	1.13 (0.66, 1.93)	1.31 (0.71, 2.40)	1.15 (0.62, 2.12)	0.38
≥80	0.73 (0.33, 1.59)	1.03 (0.43, 2.46)	0.90 (0.37, 2.20)	0.94
Sex				
Male	1.00 (reference)	1.00 (reference)	1.00 (reference)	
Female	1.62 (0.98, 2.67)	1.90 (1.12, 3.21)	1.74 (1.10, 3.02)	0.02
Education level				
No school	1.00 (reference)	1.00 (reference)	1.00 (reference)	
Primary school	2.72 (1.31, 5.64)	3.13 (1.45, 6.74)	3.03 (1.34, 6.86)	0.008
>Primary school	1.83 (1.02, 3.50)	2.22 (1.06, 4.63)	2.48 (1.31, 5.44)	0.02
Mild depression				
No	1.00 (reference)	-	1.00 (reference)	
Yes	3.22 (1.46, 7.11)	-	2.65 (1.11, 6.36)	0.03
Family relationships				
Good	1.00 (reference)	-	1.00 (reference)	
Poor	3.95 (2.22, 7.03)	-	3.65 (1.98, 6.75)	<0.001

OR, odds ratio; CI, confidence interval; aOR, adjusted OR.

1 Model I: adjusted for age, sex and education level.

2 Model II: adjusted for age, sex, education level, mild depression, and family relationships.
